# 5,5′-Dimeth­oxy-2,2′-[2,2-dimethyl­propane-1,3-diylbis(nitrilo­methyl­idyne)]diphenol

**DOI:** 10.1107/S1600536808037690

**Published:** 2008-11-20

**Authors:** Chin Sing Yeap, Hadi Kargar, Reza Kia, Hoong-Kun Fun

**Affiliations:** a X-ray Crystallography Unit, School of Physics, Universiti Sains Malaysia, 11800 USM, Penang, Malaysia; b Department of Chemistry, School of Science, Payame Noor University (PNU), Ardakan, Yazd, Iran

## Abstract

The asymmetric unit of the title Schiff base compound, C_21_H_26_N_2_O_4_, consists of four crystallographically independent mol­ecules, *viz. A*, *B*, *C* and *D*. The *A* and *D*, and the *B* and *C* mol­ecules are related by a pseudo-inversion centre, and the remaining pairs of mol­ecules differ in the orientations of one of the meth­oxy groups. In each independent mol­ecule, intra­molecular O—H⋯N hydrogen bonds generate two *S*(6) ring motifs. The dihedral angles between the benzene rings in mol­ecules *A*, *B*, *C* and *D* are 65.86 (19), 50.41 (19), 68.59 (19) and 50.85 (19)°, respectively. In the crystal structure, mol­ecules are linked by C—H⋯O hydrogen bonds, forming *R*_2_^2^(8) dimers. In addition, weak C—H⋯π inter­actions are observed.

## Related literature

For bond-length data, see: Allen *et al.* (1987 ▶). For hydrogen-bond motifs, see: Bernstein *et al.* (1995 ▶). For crystal structures of Schiff base ligands and complexes, see: Calligaris & Randaccio (1987 ▶); Li *et al.* (2005 ▶); Bomfim *et al.* (2005 ▶); Fun *et al.* (2008 ▶). Glidewell *et al.* (2006 ▶); Sun *et al.* (2004 ▶).
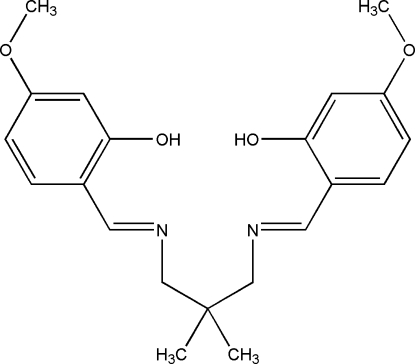


## Experimental

### 

#### Crystal data

C_21_H_26_N_2_O_4_
*M* *_r_* = 370.44
Monoclinic, 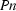

*a* = 10.2940 (2) Å
*b* = 11.8173 (2) Å
*c* = 31.5327 (5) Å
β = 93.373 (1)°
*V* = 3829.22 (12) Å^3^
*Z* = 8
Mo *K*α radiation
μ = 0.09 mm^−1^
*T* = 100.0 (1) K
0.48 × 0.13 × 0.06 mm

#### Data collection

Bruker SMART APEXII CCD area-detector diffractometer
Absorption correction: multi-scan (*SADABS*; Bruker, 2005 ▶) *T*_min_ = 0.959, *T*_max_ = 0.995
62375 measured reflections
11318 independent reflections
7933 reflections with *I* > 2σ(*I*)
*R*_int_ = 0.067

#### Refinement

*R*[*F* ^2^ > 2σ(*F* ^2^)] = 0.063
*wR*(*F* ^2^) = 0.136
*S* = 1.02
11318 reflections
989 parameters
2 restraints
H-atom parameters constrained
Δρ_max_ = 0.37 e Å^−3^
Δρ_min_ = −0.28 e Å^−3^

### 

Data collection: *APEX2* (Bruker, 2005 ▶); cell refinement: *SAINT* (Bruker, 2005 ▶); data reduction: *SAINT*; program(s) used to solve structure: *SHELXTL* (Sheldrick, 2008 ▶); program(s) used to refine structure: *SHELXTL*; molecular graphics: *SHELXTL*; software used to prepare material for publication: *SHELXTL* and *PLATON* (Spek, 2003 ▶).

## Supplementary Material

Crystal structure: contains datablocks global, I. DOI: 10.1107/S1600536808037690/ci2715sup1.cif

Structure factors: contains datablocks I. DOI: 10.1107/S1600536808037690/ci2715Isup2.hkl

Additional supplementary materials:  crystallographic information; 3D view; checkCIF report

## Figures and Tables

**Table 1:** Hydrogen-bond geometry (Å, °) *Cg*1, *Cg*2, *Cg*3 and *Cg*4 are the centroids of the C1*B*–C6*B*, C12*B*–C17*B*, C1*D*–C6*D* and C12*D*–C17*D* benzene rings.

*D*—H⋯*A*	*D*—H	H⋯*A*	*D*⋯*A*	*D*—H⋯*A*
O1*A*—H1*OA*⋯N1*A*	0.84	1.84	2.582 (4)	146
O2*A*—H2*OA*⋯N2*A*	0.84	1.87	2.621 (4)	147
O1*B*—H1*OB*⋯N1*B*	0.84	1.86	2.595 (4)	145
O2*B*—H2*OB*⋯N2*B*	0.84	1.87	2.611 (4)	147
O1*C*—H1*OC*⋯N1*C*	0.84	1.84	2.584 (5)	147
O2*C*—H2*OC*⋯N2*C*	0.84	1.86	2.607 (5)	148
O1*D*—H1*OD*⋯N1*D*	0.84	1.83	2.578 (4)	148
O2*D*—H2*OD*⋯N2*D*	0.84	1.85	2.598 (4)	148
C2*A*—H2*AA*⋯O1*C*^i^	0.95	2.55	3.426 (5)	154
C2*B*—H2*BA*⋯O1*D*^ii^	0.95	2.56	3.504 (5)	171
C2*C*—H2*CA*⋯O1*A*^iii^	0.95	2.53	3.399 (5)	151
C2*D*—H2*DA*⋯O1*B*^iv^	0.95	2.54	3.475 (5)	168
C19*C*—H19*H*⋯*Cg*1^v^	0.98	2.72	3.421 (4)	129
C19*D*—H19*K*⋯*Cg*2^vi^	0.98	2.66	3.405 (4)	133
C19*B*—H19*F*⋯*Cg*3^vii^	0.98	2.76	3.479 (4)	131
C10*B*—H10*D*⋯*Cg*4^vii^	0.99	2.81	3.803 (5)	178
C19*A*—H19*C*⋯*Cg*4^viii^	0.98	2.61	3.385 (4)	136

Symmetry codes: (i) 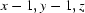; (ii) 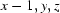; (iii) 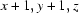; (iv) 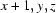; (v) 
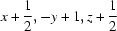
; (vi) 
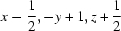
; (vii) 
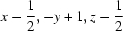
; (viii) 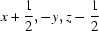.

**Fig. 1.**
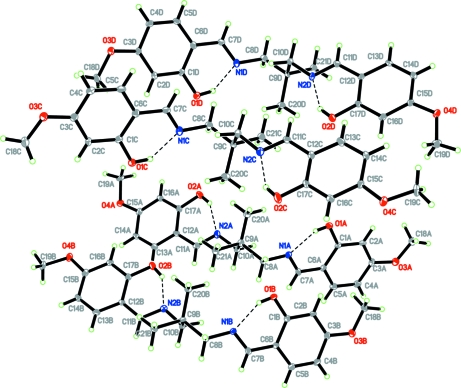
The asymmetric unit of the title compound, showing four independent molecules with atom labels. Displacement ellipsoids are drawn at the 40% probability level. Intramolecular interactions are shown as dashed lines.

**Fig. 2.**
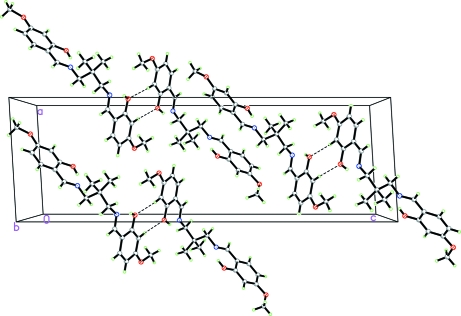
The crystal packing of the title compound, viewed down the b-axis, showing the R22(8) dimers. Hydrogen bonds are shown as dashed lines.

## supplementary crystallographic information

### Comment

In the field of coordination chemistry, Schiff base is one of most prevalent versatile ligand. The Schiff base compounds have recieved much attention due to their important role in the development of coordination chemistry related to catalysis and enzymatic reaction, magnetism, and supramolecular architectures. In comparison with Schiff-base metal complexes, only a relatively small number of free Schiff base ligands have been characterized (Calligaris & Randaccio 1987). Crystal structures of Schiff bases derived from substituted benzaldehydes and closely related to the title compound have been reported (Li *et al.*, 2005; Bomfim *et al.*, 2005; Glidewell *et al.*, 2006; Sun *et al.*, 2004).

The asymmetric unit of the title compound (Fig. 1), consists of four crystallographically independent molecules, *A*, *B*, *C* and *D*. Molecules in A/D and B/C pairs are related by pseudo-inversion centres at (0.497 0.123 0.332) and (0.486 0.627 0.331), respectively. The other pairs of molecules *viz*. A/B, C/D, A/C and B/D differ in the orientations of one of the methoxy groups (O4—C19). Bond lengths in the independent molecules are within normal ranges (Allen *et al.*,1987) and are comparable to those observed in a related structure (Fun *et al.*, 2008). In each independent molecule, intramolecular O—H···N hydrogen bonds generate two *S*(6) ring motifs (Bernstein *et al.* 1995), with the imino group being coplanar with the benzene ring. The N atoms are also in close proximity to the H atoms of dimethylpropane groups of adjacent independent molecules, with H···N distances lying in the range 2.57–2.62 Å. The dihedral angles between the two benzene rings in molecules *A*, *B*, *C* and *D* are 66.0 (2)°, 50.5 (2)°, 68.5 (2)° and 50.9 (2)°, respectively.

In the crystal structure, symmetry related A/C and B/D pairs of molecules are linked by C—H···O hydrogen bonds forming *R_2_^2^(8)* dimers (Fig. 2). The crystal structure is further stabilized by weak C—H···π interactions (Table 1).

### Experimental

In a 50 ml round-bottomed flask, 4-methoxy salicylaldehyde (2 mmol, 304 mg) was added into a 30 ml ethanolic solution of 2,2-dimethyl-1,3-propane diamine (1 mmol, 102 mg) and then the mixture was refluxed for 1 h. The resulting yellow solid was filtered and washed with cold ethanol. Single crystals suitable for *X*-ray diffraction were obtained by slow evaporation of an ethanol solution at room temperature.

### Refinement

H atoms of hydroxyl groups were constrained using a freely rotating O—H bond with a fixed distance of 0.84 Å and *U*_iso_(H)= 1.5 *U*_eq_(O). The remaining H atoms were positioned geometrically and refined using a riding model, with C—H = 0.95–0.99 Å and *U*_iso_(H)= 1.2 or 1.5 *U*_eq_(C). A rotating-group model was applied for the methoxy methyl groups.

### Crystal data

**Table:**

C_21_H_26_N_2_O_4_	*F*_000_ = 1584
*M**_r_* = 370.44	*D*_x_ = 1.285 Mg m^−^^3^
Monoclinic, *P**n*	Mo *K*α radiation λ = 0.71073 Å
Hall symbol: P -2yac	Cell parameters from 7871 reflections
*a* = 10.2940 (2) Å	θ = 2.6–28.1º
*b* = 11.8173 (2) Å	µ = 0.09 mm^−^^1^
*c* = 31.5327 (5) Å	*T* = 100.0 (1) K
β = 93.373 (1)º	Needle, yellow
*V* = 3829.22 (12) Å^3^	0.48 × 0.13 × 0.06 mm
*Z* = 8	

### Data collection

**Table:**

Bruker SMART APEXII CCD area-detector diffractometer	11318 independent reflections
Radiation source: fine-focus sealed tube	7933 reflections with *I* > 2σ(*I*)
Monochromator: graphite	*R*_int_ = 0.067
*T* = 100.0(1) K	θ_max_ = 30.2º
φ and ω scans	θ_min_ = 1.3º
Absorption correction: multi-scan(SADABS; Bruker, 2005)	*h* = −14→11
*T*_min_ = 0.959, *T*_max_ = 0.995	*k* = −16→16
62375 measured reflections	*l* = −44→43

### Refinement

**Table:**

Refinement on *F*^2^	Secondary atom site location: difference Fourier map
Least-squares matrix: full	Hydrogen site location: inferred from neighbouring sites
*R*[*F*^2^ > 2σ(*F*^2^)] = 0.063	H-atom parameters constrained
*wR*(*F*^2^) = 0.136	*w* = 1/[σ^2^(*F*_o_^2^) + (0.0499*P*)^2^ + 1.3497*P*] where *P* = (*F*_o_^2^ + 2*F*_c_^2^)/3
*S* = 1.02	(Δ/σ)_max_ = 0.001
11318 reflections	Δρ_max_ = 0.37 e Å^−^^3^
989 parameters	Δρ_min_ = −0.28 e Å^−^^3^
2 restraints	Extinction correction: none
Primary atom site location: structure-invariant direct methods	

### Special details

**Table:**

Experimental. The low-temperature data was collected with the Oxford Cyrosystem Cobra low-temperature attachment.
Geometry. All e.s.d.'s (except the e.s.d. in the dihedral angle between two l.s. planes) are estimated using the full covariance matrix. The cell e.s.d.'s are taken into account individually in the estimation of e.s.d.'s in distances, angles and torsion angles; correlations between e.s.d.'s in cell parameters are only used when they are defined by crystal symmetry. An approximate (isotropic) treatment of cell e.s.d.'s is used for estimating e.s.d.'s involving l.s. planes.
Refinement. Refinement of *F*^2^ against ALL reflections. The weighted *R*-factor *wR* and goodness of fit *S* are based on *F*^2^, conventional *R*-factors *R* are based on *F*, with *F* set to zero for negative *F*^2^. The threshold expression of *F*^2^ > σ(*F*^2^) is used only for calculating *R*-factors(gt) *etc*. and is not relevant to the choice of reflections for refinement. *R*-factors based on *F*^2^ are statistically about twice as large as those based on *F*, and *R*- factors based on ALL data will be even larger.

### Fractional atomic coordinates and isotropic or equivalent isotropic displacement parameters (Å^2^)

**Table:**

	*x*	*y*	*z*	*U*_iso_*/*U*_eq_	
O1A	0.0104 (3)	0.0218 (3)	0.30522 (10)	0.0209 (7)	
H1OA	0.0438	0.0829	0.2976	0.031*	
O2A	0.4925 (3)	0.0208 (3)	0.13526 (10)	0.0318 (8)	
H2OA	0.4382	0.0507	0.1507	0.048*	
O3A	−0.4027 (3)	−0.1648 (3)	0.29235 (11)	0.0244 (8)	
O4A	0.7043 (3)	0.0993 (2)	0.00748 (9)	0.0233 (7)	
N1A	0.0435 (4)	0.1928 (3)	0.25593 (12)	0.0185 (8)	
N2A	0.3079 (4)	0.1609 (3)	0.15505 (12)	0.0211 (8)	
C1A	−0.1088 (4)	0.0100 (4)	0.28546 (15)	0.0171 (9)	
C2A	−0.1907 (4)	−0.0722 (4)	0.29966 (15)	0.0183 (9)	
H2AA	−0.1630	−0.1190	0.3230	0.022*	
C3A	−0.3143 (4)	−0.0865 (4)	0.27978 (14)	0.0194 (9)	
C4A	−0.3542 (4)	−0.0217 (4)	0.24455 (13)	0.0231 (9)	
H4AA	−0.4367	−0.0343	0.2302	0.028*	
C5A	−0.2727 (4)	0.0606 (3)	0.23081 (13)	0.0239 (9)	
H5AA	−0.3004	0.1054	0.2069	0.029*	
C6A	−0.1494 (4)	0.0810 (4)	0.25090 (13)	0.0179 (9)	
C7A	−0.0683 (4)	0.1728 (4)	0.23793 (13)	0.0200 (9)	
H7AA	−0.0994	0.2201	0.2152	0.024*	
C8A	0.1153 (5)	0.2906 (4)	0.24241 (15)	0.0211 (10)	
H8AA	0.1372	0.3391	0.2674	0.025*	
H8AB	0.0588	0.3351	0.2221	0.025*	
C9A	0.2411 (4)	0.2600 (4)	0.22130 (13)	0.0164 (9)	
C10A	0.1992 (4)	0.1962 (4)	0.17942 (14)	0.0209 (10)	
H10A	0.1483	0.1284	0.1866	0.025*	
H10B	0.1416	0.2461	0.1615	0.025*	
C11A	0.3283 (4)	0.2153 (4)	0.12092 (14)	0.0184 (9)	
H11A	0.2755	0.2791	0.1137	0.022*	
C12A	0.4290 (4)	0.1830 (4)	0.09297 (14)	0.0182 (9)	
C13A	0.4488 (4)	0.2470 (4)	0.05653 (15)	0.0208 (9)	
H13A	0.3979	0.3131	0.0512	0.025*	
C14A	0.5390 (4)	0.2171 (4)	0.02848 (14)	0.0219 (10)	
H14A	0.5496	0.2610	0.0037	0.026*	
C15A	0.6157 (4)	0.1207 (4)	0.03686 (14)	0.0185 (9)	
C16A	0.5999 (4)	0.0553 (4)	0.07241 (14)	0.0191 (9)	
H16A	0.6517	−0.0103	0.0776	0.023*	
C17A	0.5069 (4)	0.0863 (4)	0.10073 (14)	0.0205 (9)	
C18A	−0.3709 (5)	−0.2271 (4)	0.33027 (15)	0.0254 (10)	
H18A	−0.4437	−0.2768	0.3364	0.038*	
H18B	−0.3542	−0.1745	0.3540	0.038*	
H18C	−0.2930	−0.2729	0.3265	0.038*	
C19A	0.7975 (4)	0.0121 (4)	0.01702 (13)	0.0258 (9)	
H19A	0.8646	0.0140	−0.0038	0.039*	
H19B	0.7539	−0.0617	0.0158	0.039*	
H19C	0.8379	0.0240	0.0456	0.039*	
C20A	0.3297 (5)	0.1863 (4)	0.25036 (15)	0.0244 (10)	
H20A	0.3550	0.2283	0.2764	0.037*	
H20B	0.4077	0.1660	0.2357	0.037*	
H20C	0.2831	0.1173	0.2577	0.037*	
C21A	0.3104 (5)	0.3691 (4)	0.21103 (15)	0.0272 (11)	
H21A	0.3367	0.4087	0.2375	0.041*	
H21B	0.2515	0.4175	0.1935	0.041*	
H21C	0.3876	0.3517	0.1955	0.041*	
O1B	0.0237 (3)	0.5205 (3)	0.29461 (10)	0.0185 (7)	
H1OB	0.0575	0.5816	0.2872	0.028*	
O2B	0.4426 (3)	0.4920 (3)	0.12728 (10)	0.0248 (7)	
H2OB	0.3817	0.5228	0.1396	0.037*	
O3B	−0.3984 (3)	0.3513 (3)	0.29813 (11)	0.0239 (7)	
O4B	0.7198 (3)	0.5498 (3)	0.01704 (9)	0.0245 (7)	
N1B	0.0417 (4)	0.7064 (3)	0.25147 (11)	0.0153 (7)	
N2B	0.2808 (3)	0.6524 (3)	0.14586 (11)	0.0177 (8)	
C1B	−0.1043 (4)	0.5220 (4)	0.28372 (14)	0.0159 (9)	
C2B	−0.1811 (4)	0.4342 (4)	0.29751 (14)	0.0165 (9)	
H2BA	−0.1427	0.3744	0.3140	0.020*	
C3B	−0.3143 (4)	0.4342 (4)	0.28702 (14)	0.0169 (9)	
C4B	−0.3724 (4)	0.5216 (3)	0.26296 (13)	0.0217 (8)	
H4BA	−0.4635	0.5215	0.2561	0.026*	
C5B	−0.2955 (4)	0.6084 (3)	0.24926 (12)	0.0192 (8)	
H5BA	−0.3349	0.6681	0.2330	0.023*	
C6B	−0.1607 (4)	0.6107 (3)	0.25870 (12)	0.0145 (8)	
C7B	−0.0817 (4)	0.7026 (3)	0.24353 (12)	0.0167 (8)	
H7BA	−0.1228	0.7616	0.2273	0.020*	
C8B	0.1125 (4)	0.8016 (4)	0.23531 (14)	0.0170 (9)	
H8BA	0.1419	0.8510	0.2593	0.020*	
H8BB	0.0531	0.8464	0.2160	0.020*	
C9B	0.2314 (4)	0.7652 (4)	0.21122 (14)	0.0159 (9)	
C10B	0.1783 (4)	0.6988 (4)	0.17085 (14)	0.0182 (9)	
H10C	0.1224	0.6361	0.1798	0.022*	
H10D	0.1235	0.7503	0.1526	0.022*	
C11B	0.3146 (4)	0.7064 (4)	0.11325 (14)	0.0188 (9)	
H11B	0.2717	0.7753	0.1057	0.023*	
C12B	0.4176 (4)	0.6650 (4)	0.08728 (14)	0.0159 (9)	
C13B	0.4603 (4)	0.7312 (4)	0.05423 (15)	0.0220 (10)	
H13B	0.4198	0.8023	0.0485	0.026*	
C14B	0.5593 (4)	0.6967 (4)	0.02957 (14)	0.0218 (10)	
H14B	0.5859	0.7425	0.0069	0.026*	
C15B	0.6199 (4)	0.5924 (4)	0.03885 (14)	0.0192 (9)	
C16B	0.5792 (4)	0.5246 (4)	0.07104 (14)	0.0186 (9)	
H16B	0.6200	0.4536	0.0766	0.022*	
C17B	0.4790 (4)	0.5600 (4)	0.09529 (14)	0.0186 (9)	
C18B	−0.3488 (5)	0.2615 (4)	0.32414 (15)	0.0237 (10)	
H18D	−0.4206	0.2125	0.3318	0.036*	
H18E	−0.3051	0.2926	0.3500	0.036*	
H18F	−0.2865	0.2174	0.3086	0.036*	
C19B	0.7753 (4)	0.6216 (4)	−0.01412 (12)	0.0259 (9)	
H19D	0.8464	0.5814	−0.0270	0.039*	
H19E	0.8090	0.6908	−0.0004	0.039*	
H19F	0.7081	0.6413	−0.0362	0.039*	
C20B	0.3225 (5)	0.6904 (4)	0.23865 (15)	0.0223 (10)	
H20D	0.3551	0.7327	0.2638	0.034*	
H20E	0.3959	0.6669	0.2223	0.034*	
H20F	0.2753	0.6232	0.2476	0.034*	
C21B	0.3024 (5)	0.8708 (4)	0.19819 (15)	0.0226 (10)	
H21D	0.3346	0.9121	0.2236	0.034*	
H21E	0.2427	0.9192	0.1810	0.034*	
H21F	0.3760	0.8494	0.1815	0.034*	
O1C	0.9705 (3)	0.7226 (3)	0.35880 (10)	0.0212 (7)	
H1OC	0.9287	0.6738	0.3718	0.032*	
O2C	0.5073 (4)	0.7503 (3)	0.53140 (11)	0.0352 (9)	
H2OC	0.5614	0.7169	0.5169	0.053*	
O3C	1.3828 (3)	0.9110 (3)	0.37150 (11)	0.0237 (7)	
O4C	0.2548 (3)	0.6974 (3)	0.64867 (9)	0.0248 (7)	
N1C	0.9343 (4)	0.5564 (3)	0.41005 (13)	0.0201 (8)	
N2C	0.6721 (3)	0.5944 (3)	0.51157 (12)	0.0192 (8)	
C1C	1.0901 (4)	0.7357 (4)	0.37849 (14)	0.0166 (9)	
C2C	1.1723 (4)	0.8186 (4)	0.36319 (15)	0.0182 (9)	
H2CA	1.1452	0.8635	0.3393	0.022*	
C3C	1.2939 (5)	0.8338 (4)	0.38354 (15)	0.0201 (10)	
C4C	1.3338 (4)	0.7691 (3)	0.41939 (13)	0.0238 (9)	
H4CA	1.4170	0.7811	0.4333	0.029*	
C5C	1.2515 (4)	0.6885 (4)	0.43417 (13)	0.0238 (9)	
H5CA	1.2785	0.6455	0.4586	0.029*	
C6C	1.1278 (4)	0.6685 (4)	0.41391 (13)	0.0192 (9)	
C7C	1.0469 (4)	0.5769 (4)	0.42793 (13)	0.0188 (9)	
H7CA	1.0782	0.5308	0.4510	0.023*	
C8C	0.8607 (4)	0.4586 (4)	0.42459 (15)	0.0193 (9)	
H8CA	0.9179	0.4133	0.4444	0.023*	
H8CB	0.8354	0.4103	0.3998	0.023*	
C9C	0.7372 (4)	0.4920 (4)	0.44706 (15)	0.0178 (9)	
C10C	0.7799 (4)	0.5507 (4)	0.48799 (14)	0.0189 (9)	
H10E	0.8308	0.4968	0.5063	0.023*	
H10F	0.8380	0.6143	0.4815	0.023*	
C11C	0.6401 (4)	0.5395 (4)	0.54464 (14)	0.0180 (9)	
H11C	0.6850	0.4714	0.5520	0.022*	
C12C	0.5384 (4)	0.5774 (4)	0.57091 (14)	0.0175 (9)	
C13C	0.5018 (4)	0.5129 (4)	0.60543 (15)	0.0240 (10)	
H13C	0.5433	0.4421	0.6107	0.029*	
C14C	0.4081 (4)	0.5474 (4)	0.63215 (14)	0.0208 (9)	
H14C	0.3848	0.5010	0.6551	0.025*	
C15C	0.3487 (4)	0.6518 (4)	0.62470 (14)	0.0190 (9)	
C16C	0.3822 (4)	0.7202 (4)	0.59045 (15)	0.0222 (10)	
H16C	0.3405	0.7911	0.5855	0.027*	
C17C	0.4764 (4)	0.6834 (4)	0.56401 (14)	0.0191 (9)	
C18C	1.3526 (5)	0.9732 (4)	0.33301 (15)	0.0280 (11)	
H18G	1.4259	1.0227	0.3272	0.042*	
H18H	1.2746	1.0191	0.3362	0.042*	
H18I	1.3369	0.9202	0.3094	0.042*	
C19C	0.2035 (4)	0.6263 (4)	0.68076 (13)	0.0294 (10)	
H19G	0.1359	0.6674	0.6951	0.044*	
H19H	0.2737	0.6052	0.7016	0.044*	
H19I	0.1659	0.5578	0.6675	0.044*	
C20C	0.6508 (5)	0.5671 (4)	0.41765 (15)	0.0227 (10)	
H20G	0.6966	0.6379	0.4122	0.034*	
H20H	0.5698	0.5840	0.4312	0.034*	
H20I	0.6307	0.5275	0.3907	0.034*	
C21C	0.6670 (5)	0.3807 (4)	0.45634 (16)	0.0249 (10)	
H21G	0.7238	0.3333	0.4750	0.037*	
H21H	0.6460	0.3405	0.4296	0.037*	
H21I	0.5866	0.3972	0.4703	0.037*	
O1D	0.9627 (3)	0.2368 (3)	0.36875 (10)	0.0220 (7)	
H1OD	0.9275	0.1814	0.3802	0.033*	
O2D	0.5330 (3)	0.2384 (3)	0.53526 (10)	0.0222 (7)	
H2OD	0.5880	0.2074	0.5204	0.033*	
O3D	1.3839 (3)	0.4088 (3)	0.36612 (11)	0.0242 (7)	
O4D	0.2801 (3)	0.1490 (2)	0.65354 (9)	0.0234 (7)	
N1D	0.9471 (4)	0.0482 (3)	0.40919 (12)	0.0179 (8)	
N2D	0.7056 (3)	0.0916 (3)	0.51458 (11)	0.0162 (7)	
C1D	1.0923 (4)	0.2361 (4)	0.37932 (14)	0.0154 (9)	
C2D	1.1671 (4)	0.3270 (4)	0.36582 (15)	0.0170 (9)	
H2DA	1.1280	0.3873	0.3497	0.020*	
C3D	1.2996 (4)	0.3266 (4)	0.37661 (13)	0.0179 (9)	
C4D	1.3579 (4)	0.2355 (4)	0.39994 (12)	0.0217 (8)	
H4DA	1.4491	0.2350	0.4065	0.026*	
C5D	1.2829 (4)	0.1488 (3)	0.41294 (12)	0.0202 (8)	
H5DA	1.3228	0.0884	0.4288	0.024*	
C6D	1.1482 (4)	0.1464 (4)	0.40346 (13)	0.0186 (9)	
C7D	1.0702 (4)	0.0522 (4)	0.41742 (13)	0.0171 (9)	
H7DA	1.1118	−0.0078	0.4330	0.021*	
C8D	0.8731 (4)	−0.0498 (4)	0.42389 (15)	0.0183 (9)	
H8DA	0.9317	−0.0984	0.4420	0.022*	
H8DB	0.8410	−0.0950	0.3990	0.022*	
C9D	0.7567 (4)	−0.0131 (4)	0.44929 (13)	0.0159 (9)	
C10D	0.8091 (4)	0.0483 (4)	0.48896 (13)	0.0168 (9)	
H10G	0.8648	−0.0042	0.5065	0.020*	
H10H	0.8641	0.1123	0.4806	0.020*	
C11D	0.6786 (4)	0.0337 (4)	0.54769 (13)	0.0162 (9)	
H11D	0.7270	−0.0330	0.5545	0.019*	
C12D	0.5765 (4)	0.0676 (4)	0.57480 (13)	0.0146 (8)	
C13D	0.5430 (4)	−0.0012 (4)	0.60902 (14)	0.0186 (9)	
H13D	0.5899	−0.0693	0.6147	0.022*	
C14D	0.4439 (4)	0.0278 (4)	0.63443 (14)	0.0206 (9)	
H14D	0.4222	−0.0204	0.6571	0.025*	
C15D	0.3758 (4)	0.1286 (4)	0.62660 (14)	0.0173 (9)	
C16D	0.4059 (4)	0.1999 (4)	0.59310 (14)	0.0184 (9)	
H16D	0.3589	0.2683	0.5880	0.022*	
C17D	0.5057 (4)	0.1693 (4)	0.56734 (13)	0.0144 (8)	
C18D	1.3332 (5)	0.4999 (4)	0.33981 (15)	0.0256 (11)	
H18J	1.4046	0.5499	0.3326	0.038*	
H18K	1.2697	0.5430	0.3552	0.038*	
H18L	1.2908	0.4689	0.3137	0.038*	
C19D	0.1930 (4)	0.2402 (4)	0.64351 (13)	0.0308 (10)	
H19J	0.1202	0.2373	0.6622	0.046*	
H19K	0.1597	0.2338	0.6138	0.046*	
H19L	0.2392	0.3123	0.6476	0.046*	
C20D	0.6640 (5)	0.0628 (4)	0.42166 (15)	0.0203 (9)	
H20J	0.7093	0.1325	0.4145	0.030*	
H20K	0.5876	0.0815	0.4374	0.030*	
H20L	0.6363	0.0225	0.3955	0.030*	
C21D	0.6852 (5)	−0.1219 (4)	0.46105 (15)	0.0203 (9)	
H21J	0.7444	−0.1703	0.4784	0.030*	
H21K	0.6560	−0.1623	0.4351	0.030*	
H21L	0.6097	−0.1024	0.4771	0.030*	

### Atomic displacement parameters (Å^2^)

**Table:**

	*U*^11^	*U*^22^	*U*^33^	*U*^12^	*U*^13^	*U*^23^
O1A	0.0173 (15)	0.0210 (15)	0.0240 (16)	−0.0013 (12)	−0.0019 (12)	0.0030 (12)
O2A	0.0359 (19)	0.0327 (18)	0.0284 (17)	0.0136 (14)	0.0157 (14)	0.0148 (14)
O3A	0.0203 (17)	0.0249 (17)	0.0278 (18)	−0.0048 (14)	−0.0008 (14)	−0.0001 (14)
O4A	0.0213 (14)	0.0300 (17)	0.0192 (14)	0.0067 (13)	0.0053 (11)	0.0015 (12)
N1A	0.0196 (19)	0.0165 (19)	0.0202 (19)	0.0041 (15)	0.0077 (15)	−0.0005 (15)
N2A	0.0224 (19)	0.0186 (18)	0.0231 (19)	−0.0034 (14)	0.0068 (15)	−0.0020 (14)
C1A	0.014 (2)	0.018 (2)	0.019 (2)	0.0041 (17)	−0.0003 (18)	−0.0036 (18)
C2A	0.019 (2)	0.021 (2)	0.015 (2)	0.0083 (18)	−0.0035 (17)	−0.0010 (17)
C3A	0.019 (2)	0.020 (2)	0.020 (2)	−0.0043 (17)	0.0044 (17)	−0.0061 (17)
C4A	0.0187 (19)	0.028 (2)	0.022 (2)	−0.0021 (17)	−0.0051 (16)	−0.0031 (17)
C5A	0.021 (2)	0.025 (2)	0.025 (2)	0.0027 (17)	−0.0047 (16)	0.0019 (17)
C6A	0.0158 (19)	0.023 (2)	0.0154 (19)	−0.0008 (16)	0.0017 (15)	0.0009 (16)
C7A	0.023 (2)	0.021 (2)	0.0165 (19)	0.0046 (17)	0.0088 (16)	0.0033 (16)
C8A	0.025 (2)	0.020 (2)	0.018 (2)	0.0026 (18)	0.0096 (18)	−0.0008 (17)
C9A	0.019 (2)	0.016 (2)	0.0142 (19)	−0.0040 (16)	0.0010 (15)	−0.0057 (15)
C10A	0.018 (2)	0.023 (2)	0.022 (2)	−0.0018 (17)	0.0021 (17)	0.0000 (17)
C11A	0.0182 (19)	0.016 (2)	0.021 (2)	0.0017 (15)	−0.0011 (16)	−0.0029 (16)
C12A	0.0165 (19)	0.017 (2)	0.021 (2)	−0.0005 (15)	−0.0036 (16)	0.0020 (16)
C13A	0.023 (2)	0.019 (2)	0.020 (2)	0.0042 (17)	0.0021 (17)	0.0039 (16)
C14A	0.021 (2)	0.024 (2)	0.021 (2)	0.0015 (18)	0.0011 (17)	0.0075 (17)
C15A	0.018 (2)	0.022 (2)	0.0154 (19)	−0.0034 (17)	0.0006 (16)	−0.0056 (17)
C16A	0.020 (2)	0.0143 (19)	0.023 (2)	0.0013 (15)	0.0039 (16)	0.0025 (16)
C17A	0.023 (2)	0.0154 (19)	0.023 (2)	−0.0033 (16)	0.0006 (17)	0.0049 (16)
C18A	0.028 (2)	0.025 (2)	0.024 (2)	−0.0040 (19)	0.0039 (19)	−0.0014 (18)
C19A	0.0160 (19)	0.033 (2)	0.029 (2)	0.0092 (17)	0.0001 (16)	−0.0039 (18)
C20A	0.024 (2)	0.024 (2)	0.025 (2)	−0.0019 (18)	0.0021 (19)	−0.0014 (18)
C21A	0.031 (2)	0.020 (2)	0.031 (3)	−0.0061 (19)	0.008 (2)	−0.0005 (19)
O1B	0.0126 (14)	0.0159 (14)	0.0268 (16)	0.0014 (11)	0.0004 (12)	0.0017 (12)
O2B	0.0300 (17)	0.0199 (15)	0.0257 (16)	0.0032 (12)	0.0121 (13)	0.0059 (12)
O3B	0.0199 (16)	0.0237 (17)	0.0281 (17)	−0.0044 (13)	0.0009 (14)	0.0058 (14)
O4B	0.0219 (15)	0.0343 (18)	0.0184 (14)	−0.0006 (14)	0.0096 (12)	−0.0001 (13)
N1B	0.0151 (17)	0.0167 (18)	0.0142 (17)	0.0013 (14)	0.0024 (14)	−0.0004 (14)
N2B	0.0166 (17)	0.0199 (18)	0.0165 (17)	0.0003 (13)	0.0002 (13)	−0.0016 (14)
C1B	0.016 (2)	0.016 (2)	0.016 (2)	0.0021 (17)	0.0002 (17)	−0.0011 (17)
C2B	0.016 (2)	0.021 (2)	0.0125 (19)	0.0075 (17)	0.0010 (16)	0.0009 (16)
C3B	0.018 (2)	0.0151 (19)	0.018 (2)	0.0006 (16)	0.0044 (16)	−0.0019 (16)
C4B	0.0146 (18)	0.025 (2)	0.026 (2)	0.0026 (16)	0.0024 (16)	−0.0013 (17)
C5B	0.0167 (18)	0.022 (2)	0.0192 (19)	0.0019 (15)	0.0008 (15)	0.0001 (16)
C6B	0.0139 (18)	0.0157 (19)	0.0143 (18)	−0.0002 (15)	0.0029 (14)	−0.0021 (15)
C7B	0.021 (2)	0.0163 (19)	0.0128 (18)	0.0006 (16)	0.0034 (15)	0.0001 (15)
C8B	0.020 (2)	0.017 (2)	0.015 (2)	0.0034 (16)	0.0039 (16)	0.0003 (16)
C9B	0.0108 (18)	0.0125 (19)	0.024 (2)	0.0017 (15)	−0.0009 (16)	−0.0011 (16)
C10B	0.0134 (19)	0.022 (2)	0.019 (2)	−0.0018 (16)	0.0010 (16)	−0.0043 (17)
C11B	0.0186 (19)	0.020 (2)	0.017 (2)	0.0028 (16)	−0.0035 (15)	−0.0010 (16)
C12B	0.0145 (18)	0.018 (2)	0.0155 (19)	0.0010 (15)	−0.0005 (15)	0.0018 (15)
C13B	0.023 (2)	0.022 (2)	0.021 (2)	0.0059 (17)	0.0004 (16)	0.0056 (17)
C14B	0.025 (2)	0.024 (2)	0.017 (2)	−0.0015 (18)	0.0034 (17)	0.0066 (17)
C15B	0.0165 (19)	0.026 (2)	0.0154 (19)	−0.0022 (16)	0.0022 (15)	−0.0038 (16)
C16B	0.0156 (19)	0.020 (2)	0.020 (2)	0.0024 (15)	−0.0009 (15)	−0.0007 (16)
C17B	0.019 (2)	0.018 (2)	0.019 (2)	−0.0027 (16)	−0.0012 (15)	0.0034 (16)
C18B	0.023 (2)	0.021 (2)	0.026 (2)	−0.0050 (17)	−0.0001 (18)	0.0054 (17)
C19B	0.0197 (19)	0.036 (2)	0.022 (2)	−0.0110 (17)	0.0068 (16)	−0.0032 (17)
C20B	0.023 (2)	0.020 (2)	0.024 (2)	0.0020 (18)	0.0030 (18)	0.0001 (18)
C21B	0.022 (2)	0.016 (2)	0.030 (2)	−0.0048 (17)	0.0055 (18)	−0.0014 (17)
O1C	0.0163 (14)	0.0251 (17)	0.0218 (15)	−0.0020 (12)	−0.0029 (12)	0.0028 (12)
O2C	0.046 (2)	0.0272 (17)	0.0351 (18)	0.0119 (14)	0.0252 (15)	0.0144 (14)
O3C	0.0201 (16)	0.0241 (16)	0.0267 (17)	−0.0044 (13)	−0.0004 (13)	0.0007 (13)
O4C	0.0207 (15)	0.0297 (17)	0.0249 (15)	0.0021 (13)	0.0092 (12)	0.0024 (13)
N1C	0.0224 (19)	0.0185 (19)	0.0198 (18)	−0.0031 (15)	0.0045 (15)	−0.0014 (15)
N2C	0.0193 (18)	0.0224 (18)	0.0158 (16)	−0.0029 (14)	−0.0001 (14)	−0.0006 (14)
C1C	0.016 (2)	0.020 (2)	0.014 (2)	0.0018 (18)	0.0030 (17)	−0.0045 (17)
C2C	0.019 (2)	0.017 (2)	0.019 (2)	−0.0055 (17)	0.0059 (18)	−0.0003 (17)
C3C	0.019 (2)	0.017 (2)	0.024 (2)	0.0004 (17)	0.0042 (17)	−0.0028 (17)
C4C	0.0165 (19)	0.028 (2)	0.027 (2)	0.0027 (17)	−0.0017 (16)	0.0001 (18)
C5C	0.022 (2)	0.029 (2)	0.020 (2)	0.0071 (17)	0.0008 (16)	0.0034 (17)
C6C	0.019 (2)	0.019 (2)	0.020 (2)	0.0027 (16)	0.0053 (16)	−0.0031 (16)
C7C	0.019 (2)	0.021 (2)	0.0163 (19)	0.0057 (16)	0.0001 (16)	−0.0004 (16)
C8C	0.021 (2)	0.014 (2)	0.022 (2)	−0.0031 (17)	−0.0008 (17)	−0.0017 (17)
C9C	0.019 (2)	0.016 (2)	0.0188 (19)	−0.0016 (16)	0.0036 (16)	−0.0001 (15)
C10C	0.018 (2)	0.019 (2)	0.019 (2)	−0.0036 (16)	0.0022 (16)	−0.0020 (16)
C11C	0.018 (2)	0.016 (2)	0.020 (2)	−0.0009 (16)	−0.0001 (16)	0.0016 (15)
C12C	0.019 (2)	0.018 (2)	0.0158 (19)	−0.0031 (16)	0.0029 (16)	0.0006 (15)
C13C	0.029 (2)	0.018 (2)	0.024 (2)	0.0005 (18)	0.0041 (18)	0.0034 (17)
C14C	0.023 (2)	0.023 (2)	0.0170 (19)	−0.0011 (18)	0.0051 (16)	0.0053 (16)
C15C	0.0133 (19)	0.027 (2)	0.0165 (19)	−0.0038 (16)	0.0029 (16)	0.0005 (17)
C16C	0.023 (2)	0.018 (2)	0.025 (2)	0.0030 (16)	0.0038 (17)	0.0060 (16)
C17C	0.020 (2)	0.020 (2)	0.0173 (19)	−0.0017 (16)	0.0047 (16)	0.0047 (15)
C18C	0.030 (2)	0.028 (2)	0.026 (2)	−0.009 (2)	0.003 (2)	0.003 (2)
C19C	0.031 (2)	0.034 (2)	0.025 (2)	−0.0124 (19)	0.0130 (18)	0.0011 (18)
C20C	0.021 (2)	0.026 (2)	0.020 (2)	0.0018 (18)	−0.0037 (17)	0.0001 (17)
C21C	0.026 (2)	0.018 (2)	0.030 (2)	−0.0068 (19)	0.0026 (18)	−0.0005 (18)
O1D	0.0151 (14)	0.0239 (16)	0.0268 (17)	−0.0016 (12)	0.0000 (12)	0.0045 (13)
O2D	0.0252 (16)	0.0187 (14)	0.0237 (15)	0.0046 (12)	0.0088 (12)	0.0064 (11)
O3D	0.0186 (16)	0.0260 (17)	0.0280 (17)	−0.0045 (13)	0.0009 (14)	0.0078 (14)
O4D	0.0158 (14)	0.0337 (18)	0.0214 (14)	0.0027 (13)	0.0057 (11)	0.0024 (13)
N1D	0.0207 (19)	0.0186 (19)	0.0148 (17)	−0.0026 (15)	0.0049 (15)	−0.0013 (14)
N2D	0.0161 (17)	0.0176 (17)	0.0154 (16)	−0.0024 (13)	0.0060 (13)	−0.0015 (13)
C1D	0.014 (2)	0.023 (2)	0.0102 (19)	−0.0004 (17)	0.0058 (16)	−0.0031 (17)
C2D	0.019 (2)	0.014 (2)	0.018 (2)	−0.0035 (16)	0.0044 (17)	0.0009 (16)
C3D	0.018 (2)	0.025 (2)	0.0113 (18)	−0.0038 (17)	0.0027 (15)	0.0020 (16)
C4D	0.0156 (18)	0.030 (2)	0.0187 (19)	0.0022 (17)	−0.0037 (15)	0.0024 (16)
C5D	0.022 (2)	0.023 (2)	0.0152 (18)	0.0051 (16)	0.0013 (15)	0.0022 (15)
C6D	0.021 (2)	0.022 (2)	0.0142 (19)	0.0025 (16)	0.0061 (15)	0.0005 (16)
C7D	0.019 (2)	0.018 (2)	0.0143 (19)	0.0047 (16)	0.0029 (15)	−0.0007 (15)
C8D	0.022 (2)	0.011 (2)	0.022 (2)	−0.0029 (17)	−0.0012 (18)	−0.0016 (17)
C9D	0.020 (2)	0.017 (2)	0.0112 (17)	−0.0048 (16)	0.0012 (15)	0.0014 (15)
C10D	0.0152 (19)	0.020 (2)	0.0159 (19)	0.0024 (16)	0.0064 (15)	−0.0007 (15)
C11D	0.0156 (19)	0.016 (2)	0.0168 (19)	−0.0014 (15)	−0.0008 (15)	−0.0004 (15)
C12D	0.0116 (18)	0.0176 (19)	0.0144 (18)	0.0003 (15)	−0.0009 (15)	0.0005 (14)
C13D	0.017 (2)	0.021 (2)	0.0173 (19)	0.0026 (16)	−0.0008 (16)	0.0061 (16)
C14D	0.022 (2)	0.026 (2)	0.0139 (19)	0.0002 (18)	−0.0006 (16)	0.0062 (16)
C15D	0.0125 (19)	0.027 (2)	0.0126 (18)	−0.0019 (17)	−0.0004 (15)	0.0001 (17)
C16D	0.0133 (19)	0.023 (2)	0.019 (2)	0.0004 (15)	−0.0015 (15)	0.0003 (16)
C17D	0.0146 (18)	0.0181 (19)	0.0105 (17)	−0.0011 (15)	0.0000 (14)	0.0000 (14)
C18D	0.030 (2)	0.021 (2)	0.027 (2)	−0.0068 (18)	0.0028 (19)	0.0066 (18)
C19D	0.027 (2)	0.042 (3)	0.024 (2)	0.011 (2)	0.0015 (18)	−0.0033 (19)
C20D	0.020 (2)	0.021 (2)	0.019 (2)	0.0002 (17)	−0.0044 (16)	0.0020 (16)
C21D	0.023 (2)	0.014 (2)	0.024 (2)	−0.0001 (17)	0.0043 (17)	0.0004 (16)

### Geometric parameters (Å, °)

**Table:**

O1A—C1A	1.350 (5)	O1C—C1C	1.355 (5)
O1A—H1OA	0.84	O1C—H1OC	0.84
O2A—C17A	1.351 (5)	O2C—C17C	1.350 (5)
O2A—H2OA	0.84	O2C—H2OC	0.84
O3A—C3A	1.372 (6)	O3C—C3C	1.362 (6)
O3A—C18A	1.426 (6)	O3C—C18C	1.437 (6)
O4A—C15A	1.362 (5)	O4C—C15C	1.372 (5)
O4A—C19A	1.428 (5)	O4C—C19C	1.439 (5)
N1A—C7A	1.276 (6)	N1C—C7C	1.282 (6)
N1A—C8A	1.449 (6)	N1C—C8C	1.469 (6)
N2A—C11A	1.281 (6)	N2C—C11C	1.288 (6)
N2A—C10A	1.455 (6)	N2C—C10C	1.466 (6)
C1A—C2A	1.378 (7)	C1C—C2C	1.398 (6)
C1A—C6A	1.419 (6)	C1C—C6C	1.407 (6)
C2A—C3A	1.396 (6)	C2C—C3C	1.385 (7)
C2A—H2AA	0.95	C2C—H2CA	0.95
C3A—C4A	1.391 (6)	C3C—C4C	1.405 (6)
C4A—C5A	1.372 (5)	C4C—C5C	1.374 (6)
C4A—H4AA	0.95	C4C—H4CA	0.95
C5A—C6A	1.405 (6)	C5C—C6C	1.411 (6)
C5A—H5AA	0.95	C5C—H5CA	0.95
C6A—C7A	1.442 (6)	C6C—C7C	1.450 (6)
C7A—H7AA	0.95	C7C—H7CA	0.95
C8A—C9A	1.534 (6)	C8C—C9C	1.543 (6)
C8A—H8AA	0.99	C8C—H8CA	0.99
C8A—H8AB	0.99	C8C—H8CB	0.99
C9A—C21A	1.517 (6)	C9C—C10C	1.507 (6)
C9A—C20A	1.526 (6)	C9C—C20C	1.530 (7)
C9A—C10A	1.560 (6)	C9C—C21C	1.538 (6)
C10A—H10A	0.99	C10C—H10E	0.99
C10A—H10B	0.99	C10C—H10F	0.99
C11A—C12A	1.451 (6)	C11C—C12C	1.444 (6)
C11A—H11A	0.95	C11C—H11C	0.95
C12A—C13A	1.400 (6)	C12C—C13C	1.399 (6)
C12A—C17A	1.409 (6)	C12C—C17C	1.417 (6)
C13A—C14A	1.366 (6)	C13C—C14C	1.379 (6)
C13A—H13A	0.95	C13C—H13C	0.95
C14A—C15A	1.403 (6)	C14C—C15C	1.391 (6)
C14A—H14A	0.95	C14C—H14C	0.95
C15A—C16A	1.379 (6)	C15C—C16C	1.408 (6)
C16A—C17A	1.396 (6)	C16C—C17C	1.386 (6)
C16A—H16A	0.95	C16C—H16C	0.95
C18A—H18A	0.98	C18C—H18G	0.98
C18A—H18B	0.98	C18C—H18H	0.98
C18A—H18C	0.98	C18C—H18I	0.98
C19A—H19A	0.98	C19C—H19G	0.98
C19A—H19B	0.98	C19C—H19H	0.98
C19A—H19C	0.98	C19C—H19I	0.98
C20A—H20A	0.98	C20C—H20G	0.98
C20A—H20B	0.98	C20C—H20H	0.98
C20A—H20C	0.98	C20C—H20I	0.98
C21A—H21A	0.98	C21C—H21G	0.98
C21A—H21B	0.98	C21C—H21H	0.98
C21A—H21C	0.98	C21C—H21I	0.98
O1B—C1B	1.342 (5)	O1D—C1D	1.356 (5)
O1B—H1OB	0.84	O1D—H1OD	0.84
O2B—C17B	1.360 (5)	O2D—C17D	1.343 (5)
O2B—H2OB	0.84	O2D—H2OD	0.84
O3B—C3B	1.366 (5)	O3D—C3D	1.357 (6)
O3B—C18B	1.418 (6)	O3D—C18D	1.438 (6)
O4B—C15B	1.366 (5)	O4D—C15D	1.359 (5)
O4B—C19B	1.441 (5)	O4D—C19D	1.425 (5)
N1B—C7B	1.281 (5)	N1D—C7D	1.280 (6)
N1B—C8B	1.449 (6)	N1D—C8D	1.475 (6)
N2B—C11B	1.276 (6)	N2D—C11D	1.292 (5)
N2B—C10B	1.461 (5)	N2D—C10D	1.466 (5)
C1B—C2B	1.390 (6)	C1D—C2D	1.403 (6)
C1B—C6B	1.415 (6)	C1D—C6D	1.408 (6)
C2B—C3B	1.392 (6)	C2D—C3D	1.387 (6)
C2B—H2BA	0.95	C2D—H2DA	0.95
C3B—C4B	1.396 (6)	C3D—C4D	1.417 (6)
C4B—C5B	1.380 (5)	C4D—C5D	1.360 (5)
C4B—H4BA	0.95	C4D—H4DA	0.95
C5B—C6B	1.402 (5)	C5D—C6D	1.402 (6)
C5B—H5BA	0.95	C5D—H5DA	0.95
C6B—C7B	1.455 (6)	C6D—C7D	1.455 (6)
C7B—H7BA	0.95	C7D—H7DA	0.95
C8B—C9B	1.540 (6)	C8D—C9D	1.543 (6)
C8B—H8BA	0.99	C8D—H8DA	0.99
C8B—H8BB	0.99	C8D—H8DB	0.99
C9B—C21B	1.515 (6)	C9D—C10D	1.518 (6)
C9B—C20B	1.522 (6)	C9D—C21D	1.537 (6)
C9B—C10B	1.566 (6)	C9D—C20D	1.541 (6)
C10B—H10C	0.99	C10D—H10G	0.99
C10B—H10D	0.99	C10D—H10H	0.99
C11B—C12B	1.462 (6)	C11D—C12D	1.450 (6)
C11B—H11B	0.95	C11D—H11D	0.95
C12B—C13B	1.394 (6)	C12D—C13D	1.410 (6)
C12B—C17B	1.409 (6)	C12D—C17D	1.417 (6)
C13B—C14B	1.379 (6)	C13D—C14D	1.376 (6)
C13B—H13B	0.95	C13D—H13D	0.95
C14B—C15B	1.405 (6)	C14D—C15D	1.398 (6)
C14B—H14B	0.95	C14D—H14D	0.95
C15B—C16B	1.377 (6)	C15D—C16D	1.401 (6)
C16B—C17B	1.384 (6)	C16D—C17D	1.395 (6)
C16B—H16B	0.95	C16D—H16D	0.95
C18B—H18D	0.98	C18D—H18J	0.98
C18B—H18E	0.98	C18D—H18K	0.98
C18B—H18F	0.98	C18D—H18L	0.98
C19B—H19D	0.98	C19D—H19J	0.98
C19B—H19E	0.98	C19D—H19K	0.98
C19B—H19F	0.98	C19D—H19L	0.98
C20B—H20D	0.98	C20D—H20J	0.98
C20B—H20E	0.98	C20D—H20K	0.98
C20B—H20F	0.98	C20D—H20L	0.98
C21B—H21D	0.98	C21D—H21J	0.98
C21B—H21E	0.98	C21D—H21K	0.98
C21B—H21F	0.98	C21D—H21L	0.98
			
C1A—O1A—H1OA	109.5	C1C—O1C—H1OC	109.5
C17A—O2A—H2OA	109.5	C17C—O2C—H2OC	109.5
C3A—O3A—C18A	117.9 (4)	C3C—O3C—C18C	117.5 (4)
C15A—O4A—C19A	117.4 (3)	C15C—O4C—C19C	117.2 (3)
C7A—N1A—C8A	118.7 (4)	C7C—N1C—C8C	118.7 (4)
C11A—N2A—C10A	118.0 (4)	C11C—N2C—C10C	118.1 (4)
O1A—C1A—C2A	118.7 (4)	O1C—C1C—C2C	118.3 (4)
O1A—C1A—C6A	120.8 (4)	O1C—C1C—C6C	120.0 (4)
C2A—C1A—C6A	120.5 (4)	C2C—C1C—C6C	121.7 (4)
C1A—C2A—C3A	119.9 (4)	C3C—C2C—C1C	118.7 (4)
C1A—C2A—H2AA	120.0	C3C—C2C—H2CA	120.7
C3A—C2A—H2AA	120.0	C1C—C2C—H2CA	120.7
O3A—C3A—C4A	115.6 (4)	O3C—C3C—C2C	124.2 (4)
O3A—C3A—C2A	123.7 (4)	O3C—C3C—C4C	114.8 (4)
C4A—C3A—C2A	120.7 (4)	C2C—C3C—C4C	121.0 (4)
C5A—C4A—C3A	119.0 (4)	C5C—C4C—C3C	119.5 (4)
C5A—C4A—H4AA	120.5	C5C—C4C—H4CA	120.2
C3A—C4A—H4AA	120.5	C3C—C4C—H4CA	120.2
C4A—C5A—C6A	122.2 (4)	C4C—C5C—C6C	121.4 (4)
C4A—C5A—H5AA	118.9	C4C—C5C—H5CA	119.3
C6A—C5A—H5AA	118.9	C6C—C5C—H5CA	119.3
C5A—C6A—C1A	117.5 (4)	C1C—C6C—C5C	117.7 (4)
C5A—C6A—C7A	121.6 (4)	C1C—C6C—C7C	121.8 (4)
C1A—C6A—C7A	120.9 (4)	C5C—C6C—C7C	120.5 (4)
N1A—C7A—C6A	122.4 (4)	N1C—C7C—C6C	121.9 (4)
N1A—C7A—H7AA	118.8	N1C—C7C—H7CA	119.1
C6A—C7A—H7AA	118.8	C6C—C7C—H7CA	119.1
N1A—C8A—C9A	113.5 (4)	N1C—C8C—C9C	113.3 (4)
N1A—C8A—H8AA	108.9	N1C—C8C—H8CA	108.9
C9A—C8A—H8AA	108.9	C9C—C8C—H8CA	108.9
N1A—C8A—H8AB	108.9	N1C—C8C—H8CB	108.9
C9A—C8A—H8AB	108.9	C9C—C8C—H8CB	108.9
H8AA—C8A—H8AB	107.7	H8CA—C8C—H8CB	107.7
C21A—C9A—C20A	110.0 (4)	C10C—C9C—C20C	112.4 (4)
C21A—C9A—C8A	108.2 (4)	C10C—C9C—C21C	110.3 (4)
C20A—C9A—C8A	111.3 (4)	C20C—C9C—C21C	110.4 (4)
C21A—C9A—C10A	109.9 (4)	C10C—C9C—C8C	107.7 (4)
C20A—C9A—C10A	111.0 (4)	C20C—C9C—C8C	109.6 (4)
C8A—C9A—C10A	106.4 (4)	C21C—C9C—C8C	106.1 (4)
N2A—C10A—C9A	113.7 (4)	N2C—C10C—C9C	113.9 (4)
N2A—C10A—H10A	108.8	N2C—C10C—H10E	108.8
C9A—C10A—H10A	108.8	C9C—C10C—H10E	108.8
N2A—C10A—H10B	108.8	N2C—C10C—H10F	108.8
C9A—C10A—H10B	108.8	C9C—C10C—H10F	108.8
H10A—C10A—H10B	107.7	H10E—C10C—H10F	107.7
N2A—C11A—C12A	122.4 (4)	N2C—C11C—C12C	122.5 (4)
N2A—C11A—H11A	118.8	N2C—C11C—H11C	118.8
C12A—C11A—H11A	118.8	C12C—C11C—H11C	118.8
C13A—C12A—C17A	118.2 (4)	C13C—C12C—C17C	117.5 (4)
C13A—C12A—C11A	120.0 (4)	C13C—C12C—C11C	121.0 (4)
C17A—C12A—C11A	121.8 (4)	C17C—C12C—C11C	121.4 (4)
C14A—C13A—C12A	121.9 (4)	C14C—C13C—C12C	123.0 (4)
C14A—C13A—H13A	119.0	C14C—C13C—H13C	118.5
C12A—C13A—H13A	119.0	C12C—C13C—H13C	118.5
C13A—C14A—C15A	119.0 (4)	C13C—C14C—C15C	118.4 (4)
C13A—C14A—H14A	120.5	C13C—C14C—H14C	120.8
C15A—C14A—H14A	120.5	C15C—C14C—H14C	120.8
O4A—C15A—C16A	124.4 (4)	O4C—C15C—C14C	124.9 (4)
O4A—C15A—C14A	114.5 (4)	O4C—C15C—C16C	114.2 (4)
C16A—C15A—C14A	121.1 (4)	C14C—C15C—C16C	120.9 (4)
C15A—C16A—C17A	119.4 (4)	C17C—C16C—C15C	119.5 (4)
C15A—C16A—H16A	120.3	C17C—C16C—H16C	120.2
C17A—C16A—H16A	120.3	C15C—C16C—H16C	120.2
O2A—C17A—C16A	118.5 (4)	O2C—C17C—C16C	118.6 (4)
O2A—C17A—C12A	121.1 (4)	O2C—C17C—C12C	120.7 (4)
C16A—C17A—C12A	120.4 (4)	C16C—C17C—C12C	120.7 (4)
O3A—C18A—H18A	109.5	O3C—C18C—H18G	109.5
O3A—C18A—H18B	109.5	O3C—C18C—H18H	109.5
H18A—C18A—H18B	109.5	H18G—C18C—H18H	109.5
O3A—C18A—H18C	109.5	O3C—C18C—H18I	109.5
H18A—C18A—H18C	109.5	H18G—C18C—H18I	109.5
H18B—C18A—H18C	109.5	H18H—C18C—H18I	109.5
O4A—C19A—H19A	109.5	O4C—C19C—H19G	109.5
O4A—C19A—H19B	109.5	O4C—C19C—H19H	109.5
H19A—C19A—H19B	109.5	H19G—C19C—H19H	109.5
O4A—C19A—H19C	109.5	O4C—C19C—H19I	109.5
H19A—C19A—H19C	109.5	H19G—C19C—H19I	109.5
H19B—C19A—H19C	109.5	H19H—C19C—H19I	109.5
C9A—C20A—H20A	109.5	C9C—C20C—H20G	109.5
C9A—C20A—H20B	109.5	C9C—C20C—H20H	109.5
H20A—C20A—H20B	109.5	H20G—C20C—H20H	109.5
C9A—C20A—H20C	109.5	C9C—C20C—H20I	109.5
H20A—C20A—H20C	109.5	H20G—C20C—H20I	109.5
H20B—C20A—H20C	109.5	H20H—C20C—H20I	109.5
C9A—C21A—H21A	109.5	C9C—C21C—H21G	109.5
C9A—C21A—H21B	109.5	C9C—C21C—H21H	109.5
H21A—C21A—H21B	109.5	H21G—C21C—H21H	109.5
C9A—C21A—H21C	109.5	C9C—C21C—H21I	109.5
H21A—C21A—H21C	109.5	H21G—C21C—H21I	109.5
H21B—C21A—H21C	109.5	H21H—C21C—H21I	109.5
C1B—O1B—H1OB	109.5	C1D—O1D—H1OD	109.5
C17B—O2B—H2OB	109.5	C17D—O2D—H2OD	109.5
C3B—O3B—C18B	118.2 (4)	C3D—O3D—C18D	117.5 (4)
C15B—O4B—C19B	117.9 (3)	C15D—O4D—C19D	117.7 (3)
C7B—N1B—C8B	118.1 (4)	C7D—N1D—C8D	119.2 (4)
C11B—N2B—C10B	119.1 (4)	C11D—N2D—C10D	117.3 (4)
O1B—C1B—C2B	118.5 (4)	O1D—C1D—C2D	118.1 (4)
O1B—C1B—C6B	121.1 (4)	O1D—C1D—C6D	120.2 (4)
C2B—C1B—C6B	120.3 (4)	C2D—C1D—C6D	121.8 (4)
C1B—C2B—C3B	119.8 (4)	C3D—C2D—C1D	118.2 (4)
C1B—C2B—H2BA	120.1	C3D—C2D—H2DA	120.9
C3B—C2B—H2BA	120.1	C1D—C2D—H2DA	120.9
O3B—C3B—C2B	124.7 (4)	O3D—C3D—C2D	124.8 (4)
O3B—C3B—C4B	114.3 (4)	O3D—C3D—C4D	114.5 (4)
C2B—C3B—C4B	120.9 (4)	C2D—C3D—C4D	120.7 (4)
C5B—C4B—C3B	119.0 (4)	C5D—C4D—C3D	119.9 (4)
C5B—C4B—H4BA	120.5	C5D—C4D—H4DA	120.0
C3B—C4B—H4BA	120.5	C3D—C4D—H4DA	120.0
C4B—C5B—C6B	121.8 (4)	C4D—C5D—C6D	121.5 (4)
C4B—C5B—H5BA	119.1	C4D—C5D—H5DA	119.3
C6B—C5B—H5BA	119.1	C6D—C5D—H5DA	119.3
C5B—C6B—C1B	118.2 (4)	C5D—C6D—C1D	117.9 (4)
C5B—C6B—C7B	120.7 (4)	C5D—C6D—C7D	120.5 (4)
C1B—C6B—C7B	121.1 (4)	C1D—C6D—C7D	121.6 (4)
N1B—C7B—C6B	121.9 (4)	N1D—C7D—C6D	121.6 (4)
N1B—C7B—H7BA	119.0	N1D—C7D—H7DA	119.2
C6B—C7B—H7BA	119.0	C6D—C7D—H7DA	119.2
N1B—C8B—C9B	112.8 (4)	N1D—C8D—C9D	111.9 (4)
N1B—C8B—H8BA	109.0	N1D—C8D—H8DA	109.2
C9B—C8B—H8BA	109.0	C9D—C8D—H8DA	109.2
N1B—C8B—H8BB	109.0	N1D—C8D—H8DB	109.2
C9B—C8B—H8BB	109.0	C9D—C8D—H8DB	109.2
H8BA—C8B—H8BB	107.8	H8DA—C8D—H8DB	107.9
C21B—C9B—C20B	110.0 (4)	C10D—C9D—C21D	110.7 (4)
C21B—C9B—C8B	108.3 (4)	C10D—C9D—C20D	111.5 (4)
C20B—C9B—C8B	111.3 (4)	C21D—C9D—C20D	109.5 (4)
C21B—C9B—C10B	110.0 (4)	C10D—C9D—C8D	108.2 (4)
C20B—C9B—C10B	110.2 (4)	C21D—C9D—C8D	106.7 (4)
C8B—C9B—C10B	107.0 (3)	C20D—C9D—C8D	110.1 (4)
N2B—C10B—C9B	113.5 (3)	N2D—C10D—C9D	112.7 (4)
N2B—C10B—H10C	108.9	N2D—C10D—H10G	109.1
C9B—C10B—H10C	108.9	C9D—C10D—H10G	109.1
N2B—C10B—H10D	108.9	N2D—C10D—H10H	109.1
C9B—C10B—H10D	108.9	C9D—C10D—H10H	109.1
H10C—C10B—H10D	107.7	H10G—C10D—H10H	107.8
N2B—C11B—C12B	121.6 (4)	N2D—C11D—C12D	121.9 (4)
N2B—C11B—H11B	119.2	N2D—C11D—H11D	119.1
C12B—C11B—H11B	119.2	C12D—C11D—H11D	119.1
C13B—C12B—C17B	118.0 (4)	C13D—C12D—C17D	118.0 (4)
C13B—C12B—C11B	120.1 (4)	C13D—C12D—C11D	120.6 (4)
C17B—C12B—C11B	121.8 (4)	C17D—C12D—C11D	121.3 (4)
C14B—C13B—C12B	122.1 (4)	C14D—C13D—C12D	121.6 (4)
C14B—C13B—H13B	118.9	C14D—C13D—H13D	119.2
C12B—C13B—H13B	118.9	C12D—C13D—H13D	119.2
C13B—C14B—C15B	118.4 (4)	C13D—C14D—C15D	119.4 (4)
C13B—C14B—H14B	120.8	C13D—C14D—H14D	120.3
C15B—C14B—H14B	120.8	C15D—C14D—H14D	120.3
O4B—C15B—C16B	115.3 (4)	O4D—C15D—C14D	114.6 (4)
O4B—C15B—C14B	123.8 (4)	O4D—C15D—C16D	124.4 (4)
C16B—C15B—C14B	120.9 (4)	C14D—C15D—C16D	121.0 (4)
C15B—C16B—C17B	120.0 (4)	C17D—C16D—C15D	119.1 (4)
C15B—C16B—H16B	120.0	C17D—C16D—H16D	120.5
C17B—C16B—H16B	120.0	C15D—C16D—H16D	120.5
O2B—C17B—C16B	118.5 (4)	O2D—C17D—C16D	118.3 (4)
O2B—C17B—C12B	120.9 (4)	O2D—C17D—C12D	120.8 (4)
C16B—C17B—C12B	120.5 (4)	C16D—C17D—C12D	120.8 (4)
O3B—C18B—H18D	109.5	O3D—C18D—H18J	109.5
O3B—C18B—H18E	109.5	O3D—C18D—H18K	109.5
H18D—C18B—H18E	109.5	H18J—C18D—H18K	109.5
O3B—C18B—H18F	109.5	O3D—C18D—H18L	109.5
H18D—C18B—H18F	109.5	H18J—C18D—H18L	109.5
H18E—C18B—H18F	109.5	H18K—C18D—H18L	109.5
O4B—C19B—H19D	109.5	O4D—C19D—H19J	109.5
O4B—C19B—H19E	109.5	O4D—C19D—H19K	109.5
H19D—C19B—H19E	109.5	H19J—C19D—H19K	109.5
O4B—C19B—H19F	109.5	O4D—C19D—H19L	109.5
H19D—C19B—H19F	109.5	H19J—C19D—H19L	109.5
H19E—C19B—H19F	109.5	H19K—C19D—H19L	109.5
C9B—C20B—H20D	109.5	C9D—C20D—H20J	109.5
C9B—C20B—H20E	109.5	C9D—C20D—H20K	109.5
H20D—C20B—H20E	109.5	H20J—C20D—H20K	109.5
C9B—C20B—H20F	109.5	C9D—C20D—H20L	109.5
H20D—C20B—H20F	109.5	H20J—C20D—H20L	109.5
H20E—C20B—H20F	109.5	H20K—C20D—H20L	109.5
C9B—C21B—H21D	109.5	C9D—C21D—H21J	109.5
C9B—C21B—H21E	109.5	C9D—C21D—H21K	109.5
H21D—C21B—H21E	109.5	H21J—C21D—H21K	109.5
C9B—C21B—H21F	109.5	C9D—C21D—H21L	109.5
H21D—C21B—H21F	109.5	H21J—C21D—H21L	109.5
H21E—C21B—H21F	109.5	H21K—C21D—H21L	109.5
			
O1A—C1A—C2A—C3A	−179.9 (4)	O1C—C1C—C2C—C3C	179.0 (4)
C6A—C1A—C2A—C3A	0.5 (7)	C6C—C1C—C2C—C3C	0.3 (7)
C18A—O3A—C3A—C4A	−175.1 (4)	C18C—O3C—C3C—C2C	−5.8 (7)
C18A—O3A—C3A—C2A	6.3 (6)	C18C—O3C—C3C—C4C	174.9 (4)
C1A—C2A—C3A—O3A	−178.8 (4)	C1C—C2C—C3C—O3C	179.5 (4)
C1A—C2A—C3A—C4A	2.6 (7)	C1C—C2C—C3C—C4C	−1.2 (7)
O3A—C3A—C4A—C5A	178.1 (4)	O3C—C3C—C4C—C5C	−179.9 (4)
C2A—C3A—C4A—C5A	−3.2 (7)	C2C—C3C—C4C—C5C	0.8 (7)
C3A—C4A—C5A—C6A	0.7 (6)	C3C—C4C—C5C—C6C	0.5 (6)
C4A—C5A—C6A—C1A	2.3 (6)	O1C—C1C—C6C—C5C	−177.7 (4)
C4A—C5A—C6A—C7A	−175.7 (4)	C2C—C1C—C6C—C5C	1.0 (6)
O1A—C1A—C6A—C5A	177.5 (4)	O1C—C1C—C6C—C7C	6.0 (6)
C2A—C1A—C6A—C5A	−2.9 (6)	C2C—C1C—C6C—C7C	−175.3 (4)
O1A—C1A—C6A—C7A	−4.4 (6)	C4C—C5C—C6C—C1C	−1.4 (6)
C2A—C1A—C6A—C7A	175.2 (4)	C4C—C5C—C6C—C7C	175.0 (4)
C8A—N1A—C7A—C6A	−176.6 (4)	C8C—N1C—C7C—C6C	176.9 (4)
C5A—C6A—C7A—N1A	179.7 (4)	C1C—C6C—C7C—N1C	−3.5 (6)
C1A—C6A—C7A—N1A	1.7 (6)	C5C—C6C—C7C—N1C	−179.8 (4)
C7A—N1A—C8A—C9A	−114.6 (4)	C7C—N1C—C8C—C9C	112.4 (5)
N1A—C8A—C9A—C21A	−176.8 (4)	N1C—C8C—C9C—C10C	−66.8 (5)
N1A—C8A—C9A—C20A	−55.9 (5)	N1C—C8C—C9C—C20C	55.8 (5)
N1A—C8A—C9A—C10A	65.1 (5)	N1C—C8C—C9C—C21C	175.1 (4)
C11A—N2A—C10A—C9A	−106.5 (5)	C11C—N2C—C10C—C9C	104.0 (5)
C21A—C9A—C10A—N2A	62.9 (5)	C20C—C9C—C10C—N2C	55.2 (5)
C20A—C9A—C10A—N2A	−59.0 (5)	C21C—C9C—C10C—N2C	−68.5 (5)
C8A—C9A—C10A—N2A	179.8 (4)	C8C—C9C—C10C—N2C	176.0 (4)
C10A—N2A—C11A—C12A	−176.2 (4)	C10C—N2C—C11C—C12C	177.8 (4)
N2A—C11A—C12A—C13A	−178.6 (4)	N2C—C11C—C12C—C13C	176.9 (4)
N2A—C11A—C12A—C17A	2.9 (7)	N2C—C11C—C12C—C17C	−6.1 (7)
C17A—C12A—C13A—C14A	1.1 (7)	C17C—C12C—C13C—C14C	0.8 (7)
C11A—C12A—C13A—C14A	−177.4 (4)	C11C—C12C—C13C—C14C	177.9 (4)
C12A—C13A—C14A—C15A	−1.2 (7)	C12C—C13C—C14C—C15C	−0.8 (7)
C19A—O4A—C15A—C16A	−8.3 (6)	C19C—O4C—C15C—C14C	−7.6 (6)
C19A—O4A—C15A—C14A	171.5 (4)	C19C—O4C—C15C—C16C	172.1 (4)
C13A—C14A—C15A—O4A	−178.8 (4)	C13C—C14C—C15C—O4C	−179.6 (4)
C13A—C14A—C15A—C16A	1.0 (7)	C13C—C14C—C15C—C16C	0.7 (7)
O4A—C15A—C16A—C17A	179.1 (4)	O4C—C15C—C16C—C17C	179.7 (4)
C14A—C15A—C16A—C17A	−0.7 (7)	C14C—C15C—C16C—C17C	−0.5 (7)
C15A—C16A—C17A—O2A	179.7 (4)	C15C—C16C—C17C—O2C	−179.9 (4)
C15A—C16A—C17A—C12A	0.6 (7)	C15C—C16C—C17C—C12C	0.5 (7)
C13A—C12A—C17A—O2A	−179.8 (4)	C13C—C12C—C17C—O2C	179.8 (4)
C11A—C12A—C17A—O2A	−1.4 (6)	C11C—C12C—C17C—O2C	2.7 (6)
C13A—C12A—C17A—C16A	−0.7 (6)	C13C—C12C—C17C—C16C	−0.7 (7)
C11A—C12A—C17A—C16A	177.7 (4)	C11C—C12C—C17C—C16C	−177.8 (4)
O1B—C1B—C2B—C3B	179.4 (4)	O1D—C1D—C2D—C3D	180.0 (4)
C6B—C1B—C2B—C3B	−0.7 (6)	C6D—C1D—C2D—C3D	0.5 (6)
C18B—O3B—C3B—C2B	3.6 (6)	C18D—O3D—C3D—C2D	−3.8 (7)
C18B—O3B—C3B—C4B	−177.3 (4)	C18D—O3D—C3D—C4D	175.6 (4)
C1B—C2B—C3B—O3B	178.7 (4)	C1D—C2D—C3D—O3D	−179.5 (4)
C1B—C2B—C3B—C4B	−0.4 (6)	C1D—C2D—C3D—C4D	1.2 (6)
O3B—C3B—C4B—C5B	−178.6 (4)	O3D—C3D—C4D—C5D	178.8 (4)
C2B—C3B—C4B—C5B	0.6 (6)	C2D—C3D—C4D—C5D	−1.7 (6)
C3B—C4B—C5B—C6B	0.2 (6)	C3D—C4D—C5D—C6D	0.6 (6)
C4B—C5B—C6B—C1B	−1.3 (6)	C4D—C5D—C6D—C1D	1.0 (6)
C4B—C5B—C6B—C7B	179.5 (3)	C4D—C5D—C6D—C7D	179.5 (4)
O1B—C1B—C6B—C5B	−178.6 (4)	O1D—C1D—C6D—C5D	178.9 (4)
C2B—C1B—C6B—C5B	1.5 (6)	C2D—C1D—C6D—C5D	−1.6 (6)
O1B—C1B—C6B—C7B	0.7 (6)	O1D—C1D—C6D—C7D	0.5 (6)
C2B—C1B—C6B—C7B	−179.2 (4)	C2D—C1D—C6D—C7D	180.0 (4)
C8B—N1B—C7B—C6B	−179.6 (4)	C8D—N1D—C7D—C6D	179.6 (4)
C5B—C6B—C7B—N1B	−179.6 (4)	C5D—C6D—C7D—N1D	179.9 (4)
C1B—C6B—C7B—N1B	1.2 (6)	C1D—C6D—C7D—N1D	−1.7 (6)
C7B—N1B—C8B—C9B	−129.7 (4)	C7D—N1D—C8D—C9D	127.5 (4)
N1B—C8B—C9B—C21B	−176.5 (4)	N1D—C8D—C9D—C10D	−63.1 (5)
N1B—C8B—C9B—C20B	−55.5 (5)	N1D—C8D—C9D—C21D	177.7 (4)
N1B—C8B—C9B—C10B	64.9 (4)	N1D—C8D—C9D—C20D	58.9 (5)
C11B—N2B—C10B—C9B	−98.1 (5)	C11D—N2D—C10D—C9D	100.7 (4)
C21B—C9B—C10B—N2B	66.1 (5)	C21D—C9D—C10D—N2D	−66.0 (5)
C20B—C9B—C10B—N2B	−55.3 (5)	C20D—C9D—C10D—N2D	56.2 (5)
C8B—C9B—C10B—N2B	−176.5 (4)	C8D—C9D—C10D—N2D	177.4 (4)
C10B—N2B—C11B—C12B	179.2 (4)	C10D—N2D—C11D—C12D	−178.8 (4)
N2B—C11B—C12B—C13B	−174.1 (4)	N2D—C11D—C12D—C13D	175.6 (4)
N2B—C11B—C12B—C17B	4.1 (6)	N2D—C11D—C12D—C17D	−3.2 (6)
C17B—C12B—C13B—C14B	0.1 (7)	C17D—C12D—C13D—C14D	0.6 (6)
C11B—C12B—C13B—C14B	178.4 (4)	C11D—C12D—C13D—C14D	−178.2 (4)
C12B—C13B—C14B—C15B	−1.1 (7)	C12D—C13D—C14D—C15D	−0.9 (7)
C19B—O4B—C15B—C16B	−173.9 (4)	C19D—O4D—C15D—C14D	−170.1 (4)
C19B—O4B—C15B—C14B	7.0 (6)	C19D—O4D—C15D—C16D	9.3 (6)
C13B—C14B—C15B—O4B	−179.3 (4)	C13D—C14D—C15D—O4D	−180.0 (4)
C13B—C14B—C15B—C16B	1.7 (7)	C13D—C14D—C15D—C16D	0.6 (7)
O4B—C15B—C16B—C17B	179.7 (4)	O4D—C15D—C16D—C17D	−179.5 (4)
C14B—C15B—C16B—C17B	−1.2 (7)	C14D—C15D—C16D—C17D	−0.1 (6)
C15B—C16B—C17B—O2B	−179.0 (4)	C15D—C16D—C17D—O2D	179.8 (4)
C15B—C16B—C17B—C12B	0.2 (6)	C15D—C16D—C17D—C12D	−0.2 (6)
C13B—C12B—C17B—O2B	179.5 (4)	C13D—C12D—C17D—O2D	180.0 (4)
C11B—C12B—C17B—O2B	1.2 (6)	C11D—C12D—C17D—O2D	−1.3 (6)
C13B—C12B—C17B—C16B	0.4 (6)	C13D—C12D—C17D—C16D	−0.1 (6)
C11B—C12B—C17B—C16B	−177.9 (4)	C11D—C12D—C17D—C16D	178.7 (4)

### Hydrogen-bond geometry (Å, °)

**Table:**

*D*—H···*A*	*D*—H	H···*A*	*D*···*A*	*D*—H···*A*
O1A—H1OA···N1A	0.84	1.84	2.582 (4)	146
O2A—H2OA···N2A	0.84	1.87	2.621 (4)	147
O1B—H1OB···N1B	0.84	1.86	2.595 (4)	145
O2B—H2OB···N2B	0.84	1.87	2.611 (4)	147
O1C—H1OC···N1C	0.84	1.84	2.584 (5)	147
O2C—H2OC···N2C	0.84	1.86	2.607 (5)	148
O1D—H1OD···N1D	0.84	1.83	2.578 (4)	148
O2D—H2OD···N2D	0.84	1.85	2.598 (4)	148
C2A—H2AA···O1C^i^	0.95	2.55	3.426 (5)	154
C2B—H2BA···O1D^ii^	0.95	2.56	3.504 (5)	171
C2C—H2CA···O1A^iii^	0.95	2.53	3.399 (5)	151
C2D—H2DA···O1B^iv^	0.95	2.54	3.475 (5)	168
C19C—H19H···Cg1^v^	0.98	2.72	3.421 (4)	129
C19D—H19K···Cg2^vi^	0.98	2.66	3.405 (4)	133
C19B—H19F···Cg3^vii^	0.98	2.76	3.479 (4)	131
C10B—H10D···Cg4^vii^	0.99	2.81	3.803 (5)	178
C19A—H19C···Cg4^viii^	0.98	2.61	3.385 (4)	136

Symmetry codes: (i) *x*−1, *y*−1, *z*; (ii) *x*−1, *y*, *z*; (iii) *x*+1, *y*+1, *z*; (iv) *x*+1, *y*, *z*; (v) *x*+1/2, −*y*+1, *z*+1/2; (vi) *x*−1/2, −*y*+1, *z*+1/2; (vii) *x*−1/2, −*y*+1, *z*−1/2; (viii) *x*+1/2, −*y*, *z*−1/2.
